# An Interactive Module to Teach Common Biostatistical Tests to Learners in the Health Professions

**DOI:** 10.7759/cureus.36125

**Published:** 2023-03-14

**Authors:** Patricia M Hayes, Alan Cherney, Dimitrios Papanagnou

**Affiliations:** 1 Emergency Medicine, Sidney Kimmel Medical College, Thomas Jefferson University, Philadelphia, USA

**Keywords:** epidemiology, asynchronous curriculum, self-paced learning, online module, biostatistics, undergraduate medical education

## Abstract

Biostatistics are ubiquitous in medicine, providing quantitative insights into trials and experiments that shape the healthcare field. Despite training in evidence-based medicine, medical students and residents struggle to master biostatistical concepts and apply biostatistics to appraise research. There are limited resources available for students to quickly and cost-effectively learn biostatistical tests. From this problem, a two-part biostatistical educational module was created using Rise Articulate 360® software, an interactive module platform. The study aimed to assess the effectiveness of an educational biostatistics module's ability to improve learners' knowledge and application of commonly used biostatistical tests, as well as their confidence in biostatistics.

Each part of the module contained five biostatistical test tutorials. Each biostatistical test was explained, as well as how the test was typically applied in healthcare. Knowledge acquisition, test application, and confidence regarding biostatistical tests were assessed using a pretest and a posttest. The module was completed by 33 first- and second-year medical students.

Knowledge acquisition improved from a mean of 2.41 to 3.53 (*P* <= 0.001). Participants expressed that the biostatistical educational module was easy to use and improved both their confidence and knowledge of specific biostatistical tests. Most students found that the biostatistical educational module applied to their future work.

In summary, our module was successful in exposing learners in the health professions to commonly used biostatistical tests and tests’ applications to the medical literature and their future research. Biostatistics is a pillar of medical research and education, and students' mastery of the concept will prove to be of longitudinal valuable, whether they pursue careers as clinicians and/or researchers.

## Introduction

Biostatistics is a fundamental part of medical research as they provide quantitative insights into trials and experiments that shape the healthcare landscape. Evidence-based medicine (EBM) curricula have become ubiquitous in medical education, with 142 medical schools in the United States and Canada reporting that EBM is either a required or an elective course [1] and a part of the Accreditation Council for Graduate Medical Education (ACGME) core competency list for practice-based learning and improvement (PBLI) [2]. Despite these considerations, medical students, even upon graduation, struggle with EBM knowledge [3]. These deficits are well documented in residents across a variety of specialties [4-6].

Current undergraduate medical education curricula focus on basic statistical concepts, such as sensitivity, specificity, and predictive values [7]; however, the medical literature uses significantly more advanced statistics and is growing consistently in its complexity [8-9]. Supplemental medical education and board review programs that students utilize during their didactic years also focus on basic concepts, including sensitivity, specificity, and predictive values, as these concepts are heavily represented on the United States Medical Licensing Examination (USMLE) [7]. To critically appraise medical literature and make use of the respective research in future clinical practice, students should be familiar with more commonly used advanced biostatistical tests, beyond the fundamental biostatistical concepts that are emphasized in current medical school curricula [9]. The knowledge of these tests will provide learners with both the ability to perform biostatistics and evaluate published research critically. As demonstrated by a survey conducted by Hardwicke and Goodman in 2020, <25% of authors used specialized statistical review in their published articles, highlighting the need for physicians and researchers to be able to critically evaluate published statistical research [10]. While there are deficits in statistical knowledge, students and residents have expressed the importance of understanding statistical concepts and incorporating research into their current or future practice [5,11]. The combination of a knowledge deficit compounded with growing complexity in biostatistics emphasizes an unmet need in the medical school curriculum.

Despite training in EBM, medical students and residents struggle to master biostatistical concepts and apply biostatistics to research appraisal [4-6]. In conjunction with a lack of knowledge, more complex statistics are being used in the most prestigious journals [10,12]. In addition to a knowledge gap, there are financial and time factors that exacerbate the issue. As of 2022, the average cost of medical school was $57,574 a year, highlighting an already incredible financial burden on students [13]. From a training perspective, students have a wide array of material to learn in a very short time frame, and educators have an even shorter period to instruct this content. Currently, there are few resources available to help students quickly and cost-effectively learn biostatistical tests and their respective applications. From this unique problem, a two-part biostatistical educational module was developed and tested. Our study aimed to assess the effectiveness of a self-paced educational biostatistics module (which we reference and share with the readership community) in improving learners' knowledge and application of, as well as confidence with, commonly used biostatistical tests.

## Technical report

Module

We created a module based on an extensive review of the literature [14]. The subsequent review and editing of the parts of each module were conducted by two faculty physicians, one of whom is a statistical content expert. Using Rise Articulate 360® software (a self-paced, interactive module development platform), a two-part module was created. As we did not have an instructional designer available to assist our team with the development of the module, we intentionally chose this software given its user-friendly interface. Each phase of the module contained five tutorials for five biostatistical tests commonly used in medicine, for a total of 10 biostatistical tests. Additionally, a brief introductory page was added to each part of the module to review commonly used statistical vocabulary. Each test had its chapter in its respective module. Each chapter was composed of two sections, *Definition* and *Medical Literature*. The authors reviewed the module through the lens of Specific Review Standards from the Quality Matters (QM) Higher Education Rubric, Sixth Edition, to ensure that it meets general benchmarks for quality instruction [15].

The *Definition* portion of each chapter began with a clinical vignette and/or research scenario applicable to the research test that was to be described. The *Definition* portion described the types of variables used for the statistical test, how the statistical test works, post hoc test(s) (if applicable), when to use the statistical test, important limitations or assumptions, and what the results signify. Emphasis was placed on clearly delineating differences between statistical definitions of significance and null hypotheses, as well as the practical applications of these definitions. 

The *Medical Literature* component of each chapter used an Open Access journal article to demonstrate to students a classic example of the biostatistical test being used in medical research and an explanation of why the test was intentionally used. The module included a brief *test-your-knowledge* question that referenced the journal article students had just read. Students immediately saw the answers to the *test-your-knowledge* question after completion and viewed detailed explanations for each question. Each chapter ended with a conclusion on the use of the respective test. The exact biostatistical tests that were included in each module are displayed in Table 1. 

**Table 1:** Biostatistical tests used in each phase of the biostatistical education module. The biostatistical tests included in the module are listed in the table. There are two phases, each consisting of five common biostatistical tests, for a total of 10. ANOVA, analysis of variance

Phase	Biostatistical test
Phase 1	Two-sample t-test (independent t-test)
	Paired t-test
	One-way ANOVA
	Two-way ANOVA
	Chi-square test of independence
Phase 2	Kruskal-Wallis test
	Pearson correlation and simple linear regression
	Multiple linear regression
	Simple logistic regression
	Multiple logistic regression

Implementation 

All first- and second-year medical students at an urban medical school in Philadelphia were invited to complete the module through email correspondence. All pretests were administered and completed anonymously, employing user-generated keys to keep data paired. Participants were emailed the pretest, and upon successful completion of the pretest, they were able to access the link to the biostatistical educational module.

The module was created so that each preceding portion of the module had to be completed before participants were able to proceed to the subsequent portion. After all sections of the respective chapter were completed, participants were permitted by the software to move on to the following section of the module. Following the completion of the final section of the module, the participants were able to complete the posttest.

The pretest consisted of two components, the first of which assessed participants’ baseline knowledge of biostatistical tests through a multiple-choice question (MCQ, knowledge check) exam. The participants were required to select the appropriate biostatistical test when presented with a specific research scenario (application check). The second portion of the pretest asked the participants to report their familiarity, confidence with the application, and comprehension of biostatistical tests using a five-point Likert scale (1 = strongly disagree, 5 = strongly agree; confidence check).

The posttest was composed of three components. The first two components were identical to the pretest: the MCQ items regarding knowledge of biostatistical tests, as well as questions gauging familiarity, confidence with the application, and comprehension of biostatistical tests, were again included, using a similar five-point Likert scale (1 = strongly disagree, 5 = strongly agree). A final component was added to the posttest as the participants were asked to report their satisfaction with various aspects of the module, also employing a five-point Likert scale. 

Statistical Analysis

Statistical analysis was completed using IBM SPSS Statistics for Macintosh, Version 29 (IBM Corp., Armonk, NY, USA). Pretest and posttest MCQ scores were analyzed using a two-tailed paired t-test. Pretest and posttest Likert statements were analyzed using Wilcoxon signed-rank tests to account for nonparametric data and presented as means with standard deviations (SDs). The posttest module satisfaction results were analyzed using descriptive statistics.

Results

Forty-three first- and second-year medical students completed the pretest. Thirty-three first- and second-year medical students completed the posttest. Ten students did not complete the posttest. One student only completed the posttest module satisfaction component and was, therefore, only included in the data regarding the module's satisfaction; this student’s data was not included in any pre- and posttest comparisons.

Participants were asked to evaluate their confidence, application, and understanding of biostatistics in the pretest and posttest. The participants reported significant improvement in four of the five areas regarding confidence with biostatistics, biostatistical tests, and understanding of the application of tests by study researchers (Table 2). The participants, however, did not report an improvement in understanding how biostatistical data relates to clinical outcomes. No items were reduced in confidence or understanding (Table 2).

**Table 2:** Pretest and posttest means of medical students' confidence, usage, and familiarity of biostatistics and biostatistical tests on a Likert scale. Statements assessed on a five-point Likert scale: 1 = Strongly Disagree, 2 = Somewhat Disagree, 3 = Neither Agree nor Disagree, 4 = Somewhat Agree, and 5 = Strongly Agree. ^*^Significance established using Wilcoxon signed-rank test (*P* < 0.05).

Likert scale statement	Pretest, *n *= 32 (mean ± SD)	Posttest, *n *= 32 (mean ± SD)	*Z*-value	*P*-value
I have confidence in my ability to decide on a biostatistical test to use for my research.	2.53 ± 1.22	3.97 ± 0.65	-4.131	<0.001^*^
When reading medical literature, I understand why the researcher(s) used the biostatistical tests they did.	2.72 ± 1.35	3.94 ± 0.84	-3.984	<0.001^*^
When reading medical literature, I am sure about how the biostatistical data relates to clinical outcomes.	2.75 ± 1.20	2.81 ± 1.26	-0.015	0.988
I am familiar with several biostatistical tests.	2.94 ± 1.24	4.34 ± 0.66	-4.173	<0.001^*^

On the knowledge assessment portion of the pretest and posttest, learners demonstrated a significant improvement in knowledge from a mean of 2.41 to 3.53 (Table 3). 

**Table 3:** Paired samples t-test on pretest and posttest MCQ students’ scores. ^*^Significance established using paired samples *t*-test (*P*  < 0.05). MCQ, multiple-choice question; Ci, confidence interval; LL, lower limit; UL, upper limit

		N	Mean	Paired differences					t	df	*P*-value (Two-tailed)
				Mean difference	SD	SE	95% CI of the difference				
							LL	UL			
Score on MCQ ^a ^test	Pretest	32	2.41	-1.125	1.737	.307	-1.751	-0.499	-3.664	31	<0.001*
	Posttest	32	3.53								

**Figure 1:**
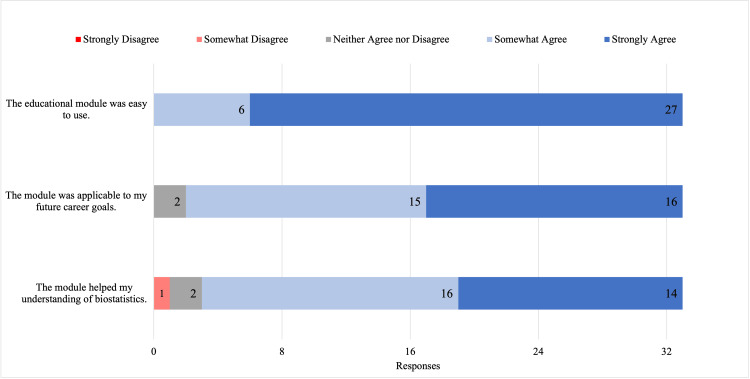
Postmodule satisfaction responses to Likert scale statements (N = 33). Students' responses to three statements regarding their satisfaction with the module.

Postmodule satisfaction responses indicated overall favorable responses regarding various aspects of the module (Figure 1). Of the 33 students, 16 (48.8%) strongly agreed and 15 (45.4%) somewhat agreed that the module was applicable to their future careers. Only two (6.0%) were neutral about its applicability to their future careers. All students either strongly agreed or somewhat agreed about the module’s ease of use (Figure 1).

## Discussion

To address the lack of curriculum available to teach learners in the health professions biostatistical concepts and their research application, we created a self-paced, two-part, biostatistical education module. The module was successful in exposing participants to commonly used biostatistical tests and tests’ applications to medical literature and their future research. We also demonstrated that using an online education module is a feasible, fast, and cost-efficient way to teach biostatistics to learners.

Most of the participants who completed the module indicated they were relatively unfamiliar with biostatistical tests or their applications. The pretest MCQ mean score was 2.41 out of five questions. These findings suggest a knowledge gap in our learner population. Our module was relevant and applicable to the intended audience, as the module continually highlighted the use of biostatistical tests in medical research and medical literature. The module required learners to read a linked journal article that used the respective biostatistical test of interest and then to answer a question about the application of the test before being able to proceed. This reinforced the *application element* of the module and allowed students to directly engage with the material. As future clinicians, students will be reading countless articles regardless of their intended specialty that utilizes these common biostatistical tests to validate their results. 

Participants’ reviews of the module were overwhelmingly positive. Most found the module easy to use, applicable to their future careers, and improved their understanding of biostatistics. Learners were shown how researchers use these statistical tests to further medical advancement and, thus, how they can employ these statistics when completing their own current and future research projects and clinical studies. However, the learners indicated that the module was not effective at teaching how biostatistical data relates to clinical outcomes. This emphasizes a future area of improvement, a need in the area of biostatistical education, and a potential future educational module that could be developed. In the future, modules that aim to instruct learners about the impact of data on clinical outcomes and provide even more substantial insights into practical applications of statistical significance will help address the knowledge gap. Another opportunity for further intervention would be to add sections to the module about how to compute the biostatistical tests in programs like SPSS or SAS software (SAS Institute, Cary, NC, USA). This would allow learners to have a greater ability to perform their statistics using modern computer-based programs frequently utilized in the research community.

There are several limitations to the module, and its evaluation is worthy of discussion. The module’s goal was to provide a broad foundational basis for biostatistical tests; thus, it did not delve deeply into the statistical or mathematical nuances of these tests. Additionally, the module did not instruct the participants on how to run the calculations behind the biostatistical tests. While this is beyond the scope of the module, it may be advantageous to add a section or embed existing links to resources that would allow learners to perform calculations independently. Regarding the evaluation of the module, the sample size is small because participants self-selected to participate in the module. It would be helpful, in the future, to expand the sample size to further validate the effectiveness of the module. It would also have been interesting to better understand why students self-selected to participate and clarify if their participation had to do with using the technology itself or their self-perceptions of their biostatistical knowledge. 

Participants were not followed in the following months, and therefore, there is a lack of information about how this knowledge will decay over time. Finally, the interface the module was created on did not afford us the ability to track learners' progress. Should educators be interested in this capability, the module can potentially be embedded in many learning management systems (LMS) to track students' progress and completion. Should this be of interest, there is an opportunity for other institutions to leverage LMS integration tools to extract learners' data and evaluate students' performance during module completion through the assistance of an instructional designer.

The biostatistical education module has a potential use outside of the standard medical education curriculum. This module can be used by current interns and residents as a way to rapidly review commonly used biostatistical tests they will encounter while appraising medical literature and/or conducting research during residency training. Physicians are constantly exposed to new research, and before adhering to the new research, the physicians must critically evaluate the article, including its statistics, to ensure empirical validity and safety. Our research suggests that an online module may be an effective educational format to teach basic biostatistical skills and concepts.

## Conclusions

Biostatistics is a vital component of the health professions training that can be difficult for learners to both understand and apply. Our study demonstrates that an interactive educational module is an effective method to educate learners on commonly used biostatistical tests and their applications. The module allowed our participants to quickly learn biostatistical tests and build a working foundation of knowledge through which they can approach statistics in their future research. This module begins to address the knowledge gap regarding biostatistics that exists in medicine by educating current trainees during the pre-clerkship years. However, there are still knowledge deficits in the comprehension and application of biostatistical tests among practicing physicians and physician scientists. Our results show that using an online interactive module, which we openly share with the readership of this report, may be an effective method to teach biostatistical concepts and applications to learners in the health professions. 
